# In Reply to Daungsupawong and Wiwanitkit

**DOI:** 10.1016/j.adro.2024.101511

**Published:** 2024-06-14

**Authors:** Fabio Dennstädt, Janna Hastings, Paul Martin Putora, Erwin Vu, Galina Fischer, Krisztian Süveg, Markus Glatzer, Elena Riggenbach, Hông-Linh Hà, Nikola Cihoric

**Affiliations:** a Department of Radiation Oncology, Kantonsspital St. Gallen, St. Gallen, Switzerland; b Department of Radiation Oncology, Inselspital, Bern University Hospital and University of Bern, Bern, Switzerland; c School of Medicine, University of St. Gallen, St. Gallen, Switzerland; d Institute for Implementation Science in Health Care, University of Zurich, Zurich, Switzerland

We thank the colleagues from Laos and India for their valuable comment on our work. Due to the considerable advantages in the field and the emerging new capabilities of large language models (LLMs), there is increasing interest in developing and applying such models in medicine.^1^ Powerful LLMs optimized for application in the clinical context may support clinicians in daily life in the future. Radiation oncology, as a data-driven and technical medical discipline in the first place, may be particularly suitable for using these new technologies. To properly contextualize the role of this technology in the clinic and to recognize potential weaknesses and dangers of evaluation studies are of great importance. Our work is one of several new studies evaluating modern LLMs (in particular, ChatGPT) for different questions in radiation oncology. Other examples would include the works of Huang et al,^2^ Floyd et al,^3^ or Holmes et al.^4^ For doing such evaluations, the answers of an LLM need to be compared with some sort of ground truth. Many studies done so far (including our own in part one of the evaluation) have used frameworks such as multiple-choice tests in which a clear correct or incorrect answer can be identified. However, if LLMs are indeed to be used for supporting clinicians in daily life, they have to give useful answers not only in standardized medical tests, but also to open-ended and real-life questions occurring in clinical practice. Evaluating the performance in these situations is more challenging because there is usually not a single correct answer to each question. In part 2 of our evaluation, the quality of the LLM's answer was evaluated by independent radiation oncologists. Although this approach can give some impression of an answer's quality, the factual correctness and usefulness of an answer are not necessarily determined this way, as it rather resembles a democratic consensus among domain experts. The limitations of our study are discussed in more detail in our article. As you mentioned, the subjectivity of the evaluation is a notable limitation. Assessing the quality of answers to relevant medical questions about topics of limited knowledge remains an unresolved issue. We also agree on your point that the sample size of questions used in our exploratory study is not sufficient to provide a comprehensive picture on the subject. Further studies investigating and evaluating LLMs in medicine and radiation oncology are surely needed, and we are participating in ongoing trials with the International Society for Radiation Oncology Informatics (ISROI). More research on the subject is urgently needed as these technologies are increasingly being used by clinicians and patients.^1^^,^^5^

## Disclosures

Dr Cihoric is a technical lead for the SmartOncology project and medical advisor for Wemedoo AG, Steinhausen AG, Switzerland. The authors declare no other conflicts of interest.
